# Does Presence of Femoral Arterial Calcification Have an Effect on Postoperative Complication and Mortality in Patients With Hip Fracture?

**DOI:** 10.7759/cureus.14878

**Published:** 2021-05-06

**Authors:** Fevzi Birişik, Yücel Bilgin, Serkan Bayram, Yusuf Öztürkmen

**Affiliations:** 1 Orthopaedics and Traumatology, Istanbul Training and Research Hospital, Istanbul, TUR; 2 Orthopaedics and Traumatology, Istanbul University School of Medicine, Istanbul, TUR; 3 Orthopaedics, Istanbul University, Istanbul, TUR

**Keywords:** arterial calcification, hip fracture, complication, mortality, geriatric injuries

## Abstract

Introduction

In this study, we investigated the relationship between femoral arterial calcification on preoperative hip radiography and post-operative complications and all-time mortality rates in patients with hip fracture >65 years old.

Methods

This retrospective study was conducted by evaluating the records of patients who were operated for hip fractures. All patients were divided into two groups according to the presence of lower extremity arterial calcification (LEAC) at the femoral artery which was diagnosed using the hip radiograph. The patients with and without the presence of LEAC were assigned as groups 1 and 2, respectively. A multivariate Cox algorithm was applied to recognize whether this radiological factor is independently associated with survival.

Results

The study included 530 patients (540 hips; 191 males and 339 females) with an average age of 80.8 ± 7.6 years. In the study after 24.8±19.9 months (range:0-65 months) follow-up period, 336 (63.3%) patients had expired. Conversely, 194 (36.7%) patients are surviving. Survival rates at one month and one year after surgery were 89.5% and 65.7%, respectively. 234 of 540 hips (230 patients) have LEAC on the femoral artery. The survival rate at one month, one year, and overall survival were significantly higher in patients with LEAC. The postoperative infection rate was also two times higher in patients with LEAC than without LEAC (p = 0.021). Multivariate analysis demonstrated that age, treatment modality (hemiarthroplasty), and the presence of femoral arterial calcification were independently associated with poor overall survival.

Conclusions

In our study, we found that the presence of femoral arterial calcification on the affected side of the hip identified on hip radiograph was independently associated with poor one month, one year, and overall survival as the patients had 1.5 times higher mortality rate. Additionally, a significant correlation was found between age and survival of patients with hip fracture, especially patients >80 years old.

## Introduction

Hip fracture continues to be an important health problem in line with the increase in the elderly population worldwide. It is predicted that the annual worldwide incidence of hip fractures will be more than six million by 2050 [1]. Cumulative one-year mortality rates after hip fractures are reported to be between 5.9% and 50% [2]. Older age, male gender, high American Academy of Anesthesiology (ASA) score, and prolonged-time from fracture to surgery have been reported as factors affecting mortality in hip fracture patients. [3,4].

Arterial calcification is one of the independent predictor factors of vascular morbidity and mortality in the elderly population [5]. It has been reported that if there is a significant association between fragility fractures and vascular calcification, it can be explained by the fact that these vessels and bones are a dynamic process related to the physiological microenvironment [6]. Local and systemic osteogenic factors that may be released from calcified atherosclerotic lesions may affect bone homeostasis [7]. It has been reported that the main effect of vascular calcification in bone homeostasis is osteoporosis and the development of fragility fractures due to decreased bone turnover [8]. For example, aortic calcification may contribute independently to the development of osteoporosis in the proximal femur [9].

In this study, we investigated the relationship between femoral arterial calcification on preoperative hip radiography and post-operative complications and all-time mortality rates in patients with hip fracture older than 65 years old.

## Materials and methods

For our study, ethics committee approval was obtained from "Istanbul Education and Research Hospital Clinical Research Local Ethics Committee" with the decision number 2583/2020. This retrospective study was conducted by evaluating the records of 530 patients (540 hips) for operated for hip fractures in our hospital between January 2015 and December 2018.

Inclusion criteria

Patients diagnosed with acute hip fracture (femoral neck fracture and pertrochanteric fracture), age above 65 years, minimum one-year follow-up (for surviving patients), low-energy fractures, and available demographics and medical records.

Exclusion criteria

Pathological fractures, periprosthetic fractures, high energy trauma, history of polytrauma and history of previous hip surgery.

**Figure 1:**
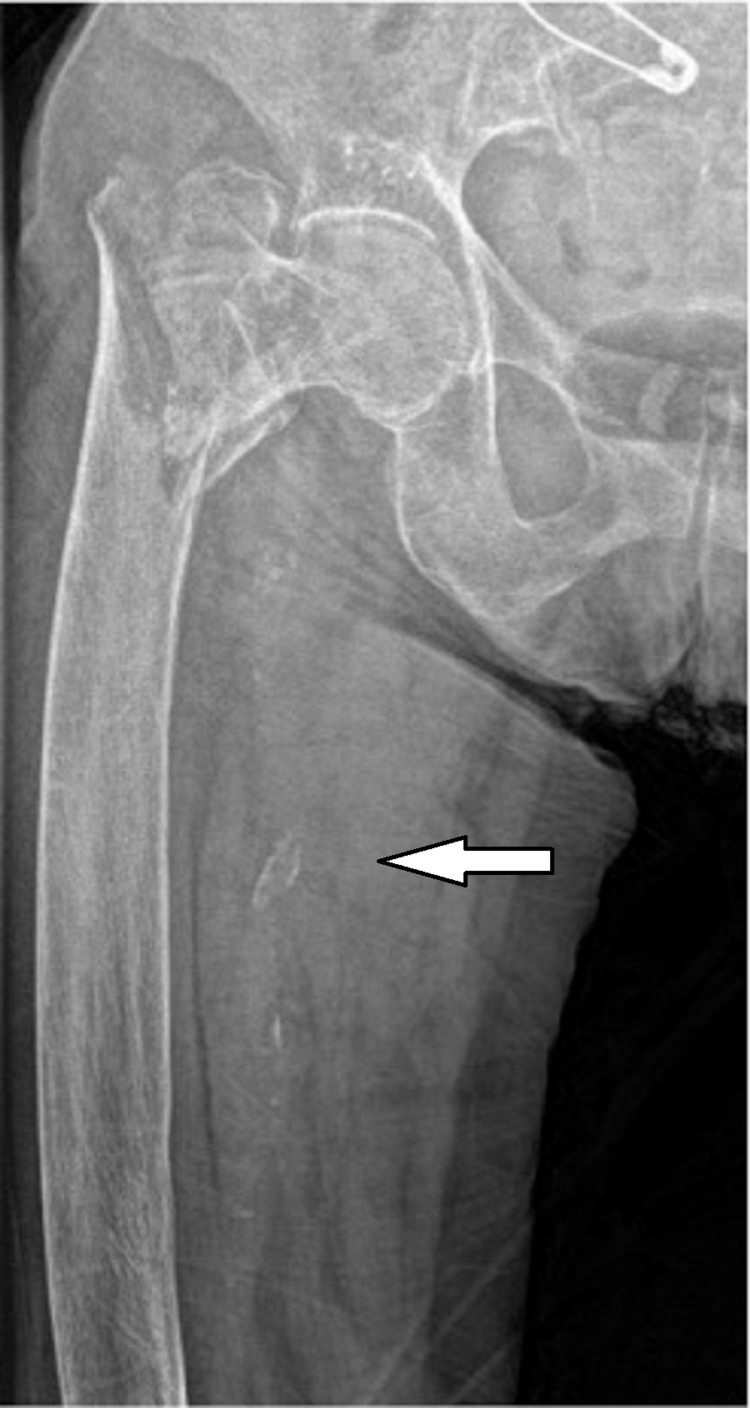
Radiographic image of the intimal type femoral artery calcification.

**Figure 2:**
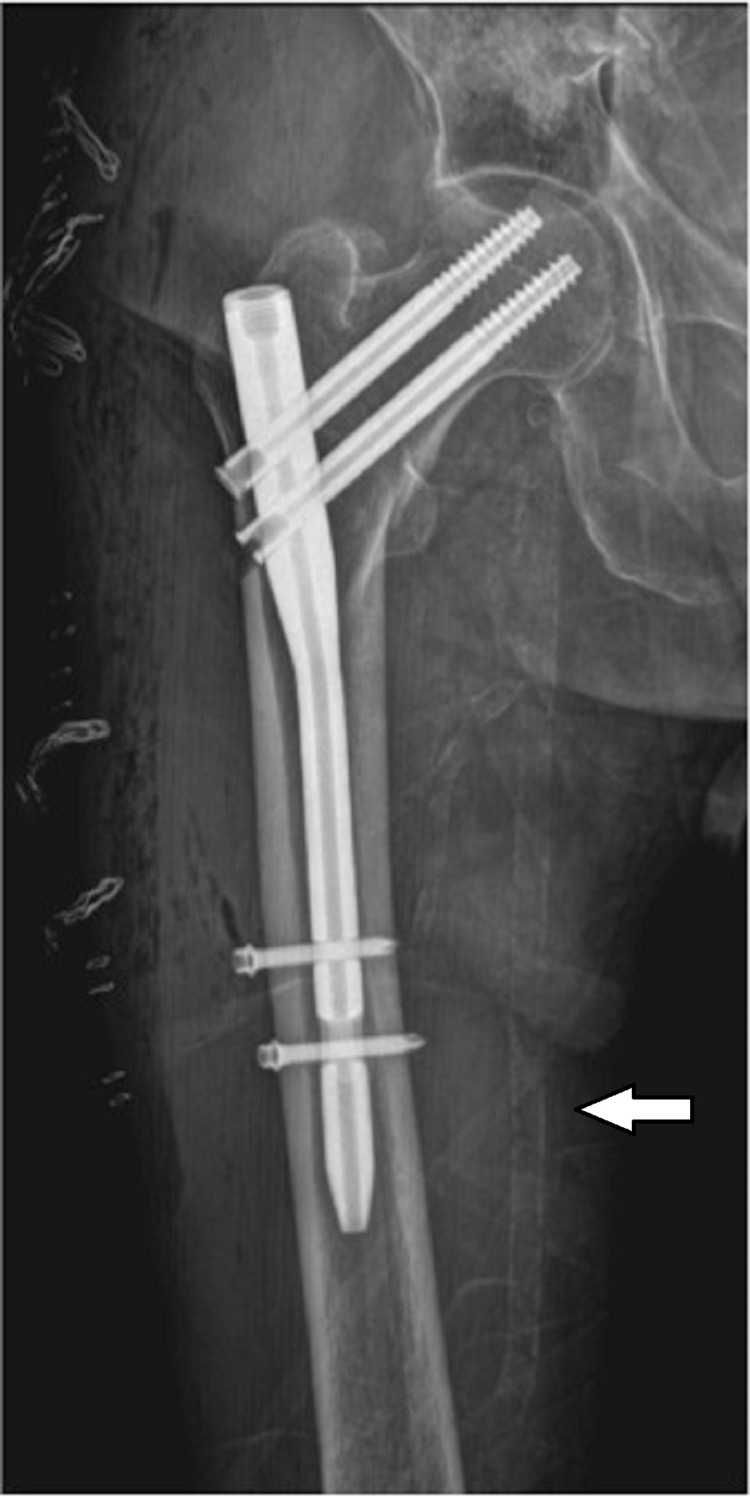
Radiographic image of the medial type femoral artery calcification.

All patients were divided into two groups according to the presence of lower extremity arterial calcification (LEAC) at femoral artery which was diagnosed at the pelvic radiograph (Figures 1, 2). The group with LEAC detected in the patients was named as group 1 and the group without LEAC was named as group 2. Calcifications in the femoral artery of the affected hip were classified as intimal, medial, or mixed [10,11].

The Social Security Administration Death Master File (Social Security Death Index) was used to determine death and the date of death of the patients. Survival of patients was also assessed at first month, first year and overall survival. All patients were operated either with intramedullary nailing, hemiarthroplasty or dynamic hip screw according to age, functional level, bone quality and the type of the fracture.

Demographic data comprised of the following variables age, gender distribution, side of fracture, type of fracture, type of treatment modality (intramedullary nailing, hemiarthroplasty or dynamic hip screw), time to surgery, hospital stay, days spent in intensive-care, type of anesthesia (regional or general), presence of postoperative surgical site infection and presence of any postoperative complication. All patients’ comorbidities as per the American Society of Anesthesiologists (ASA) score was also collected [12]. 

Statistical analysis

Statistical analysis was performed using Statistical Package for Social Sciences® (IBM Corp. Released 2016. IBM SPSS Statistics for Windows, Version 24.0. Armonk, NY: IBM Corp.) program. Descriptive statistical methods (mean, standard deviation, median, frequency, ratio, minimum, maximum) were used to evaluate the study data. The suitability of the quantitative data for normal distribution was tested by Kolmogorov-Smirnov, Shapiro-Wilk test, and graphical evaluations. Pearson chi-square test, Fisher Freeman Halton Exact test, and Fisher’s Exact test were used to compare qualitative data and Student's t-test was used for comparison of two groups of quantitative data. Kaplan-Meier survival analysis was used to evaluate the survival of the patients. Post-operative one-month and one-year mortality rates were compared using the Kaplan-Meier method. A p-value of less than 0.05 was accepted as statistically significant. . Prognostic factors were revealed in the univariate regression analysis. Elements with p values (two-sided) of 0.1 or less were included in the multivariate Cox model to identify independent variables in a stepwise fashion. Variables with p values ≤ 0.05 in the multivariate analysis were retained as independent risk factors. Receiver operator characteristics curve (ROC) analysis was performed to obtain the optimal values for the age.

## Results

**Figure 3:**
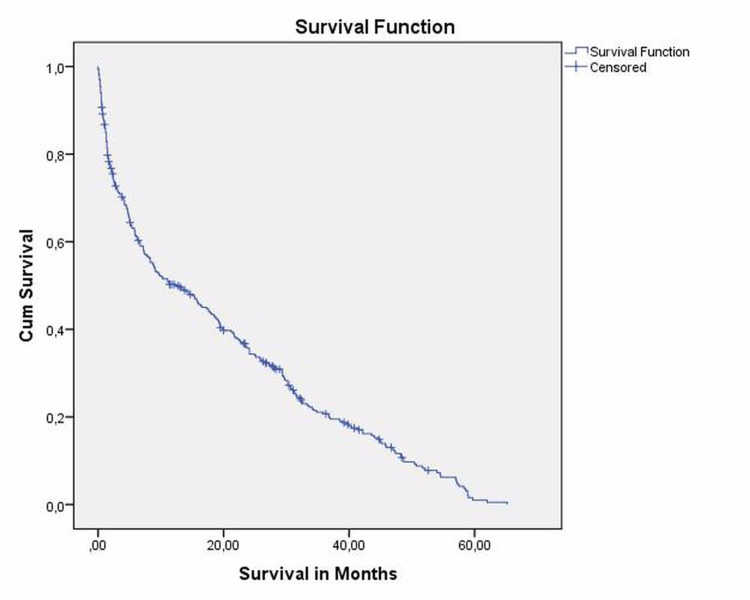
Kaplan-Meier survival analysis chart.

A total of 530 patients (540 hips) (191 male and 339 female) were included with an average age of 80.8 ± 7.6 (range: 65-102) years. At the time of this study, 336 (63.3%) patients had deceased (Figure 3). There were 194 (36.7%) surviving patients. Survival rates at first month and first year after surgery were 89.5% and 65.7%, respectively. The mean survival period following surgery was 17.1 ± 15 months (range: 0-65). There were 295 (54.6% pertrochanteric fracture and 245 (45.4%) collum fracture). Two hundred and thirty-eight hips were operated with intramedullary nailing, 280 hips with arthroplasty and 22 hips with DHS. Two hundred and thirty-four of 540 hips (230 patients, 43.3% of hips) had LEAC on femoral artery.

**Figure 4:**
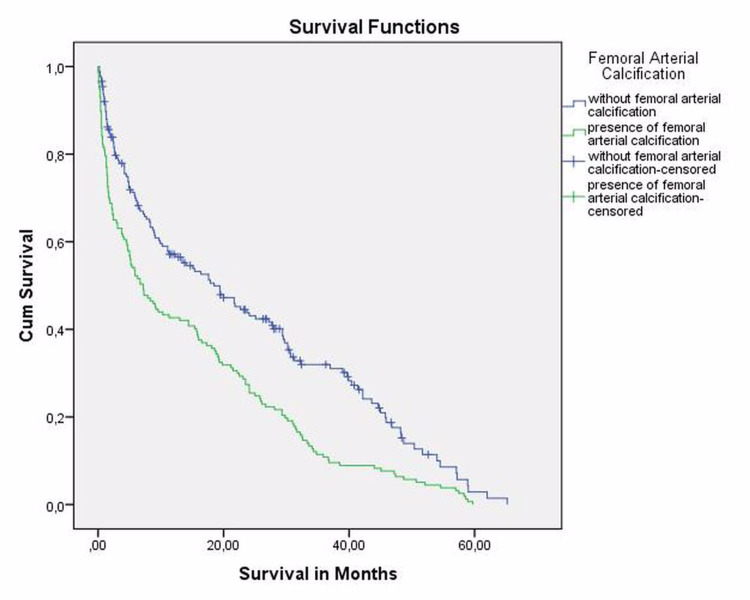
Kaplan-Meier survival analysis graph by groups.

Baseline demographic and clinical results in both groups are demonstrated in Table 1. There was a significant difference in age between group 1 and group 2 (p < 0,001). Survival rate at first month, first year, and overall survival were significantly lower in patients with LEAC (Figure 4). The mean survival after surgery was also significantly higher in patients without LEAC - 14.9 ± 16 in group 1 and 18.83 ± 17 (p = 0.042). The postoperative surgical site infection rate was also two times higher in patients with LEAC than without LEAC (p: 0.021). One hundred and forty-two hips of 234 in group 1 had intimal type LEAC and 92 hips had medial type LEAC.

**Table 1:** Comparison of survival and clinical results of both groups. *significant difference; **highly significant difference. SD: standard deviation; Min: minimum; Max: maximum; R: right; L: left; Per: pertrochanteric fracture; C: collum fracture; IMN: intramedullary nailing; HA: hemiarthroplasty; DHS: dynamic hip screw; R: regional anesthesia; G: general anesthesia; ASA: American Academy of Anesthesiology; LEAC: lower extremity arterial calcification.

	Patients with LEAC (n: 230; hip: 234)		Patients without LEAC (n: 300; hip: 306)		
	Mean ± SD	Min-max	Mean ± SD	Min-max	p-value
Age, years	82.4 ± 7.3	66-98	79.6± 7.6	65-102	<0.001**
Gender, F/M	136/94		203/97		0.061
Mortality					
In first month (N), %	37 (16.1)		19 (6.3)		<0.001**
In first year (N), %	93 (40.4)		89 (29.7)		0.010*
Overall (N) %	158 (68.7)		178 (59.3)		0.029*
Survival, months	14,9 ± 16	0-59	18.83 ± 17	0.2-65	0.042*
Side, R/L	105/129		145/161		0.602
Type of fracture, Per/C	123/111		172/134		0.433
Treatment method IMN/HA/DHS	104/124/6		134/156/16		0.297
Time to surgery, day	4.8 ± 3	1-21	4.1 ± 2.5	0-25	0.032*
Hospital stay, day	10.6 ± 8.3	2-60	8.5 ± 3.5	4-29	0.02*
Intensive care, day	1.79 ± 4	0-38	1.02 ± 2.1	0-16	0.051
ASA score					0.006*
ASA 1	9		23		
ASA 2	55		113		
ASA 3	152		149		
ASA 4	12		15		
Type of anesthesia R,G	196/38		254/52		0.408
Postoperative surgical site infection (N)	16		8		0.021*
Postoperative complication (N)	28		23		0.102

Prognostic factor analyses in patients at first month

Univariate analysis demonstrated that age (p: 0.015), hospital stay (p: 0.021), intensive care unit stay (p: 0.001) and presence of femoral arterial calcification (p < 0.001) were associated with worse overall survival. Multivariate analysis demonstrated that the presence of femoral arterial calcification (HR, 2.602 [95% CI, 1.391 to 4.866], p: 0.014) was independently associated with poor first-month survival.

Prognostic factor analyses in patients at first year

Univariate analysis demonstrated that age (p: 0.008), hospital stay (p: 0.044), and presence of femoral arterial calcification (p < 0.001) were associated with a worse overall survival. Multivariate analysis demonstrated that presence of femoral arterial calcification (HR, 1.749 [95% CI, 1.246 to 2.457], p: 0.014) was independently associated with poor first-year survival.

Prognostic factor analyses for all patients

Univariate analysis demonstrated that age (p: 0.001), treatment modality (hemiarthroplasty) (p: 0.032), and presence of femoral arterial calcification (p < 0.001) were associated with a worse overall survival. Multivariate analysis demonstrated that age HR, 1.022 [95% CI, 1.006 to 1.039], p: 0.007), treatment modality (hemiarthroplasty) HR, 0.726 [95% CI, 0.569 to 0.926], p: 0.010) presence of femoral arterial calcification (HR, 1.506 [95% CI, 1.183 to 1.916], p: 0.001) were independently associated with a poor overall survival.

**Figure 5:**
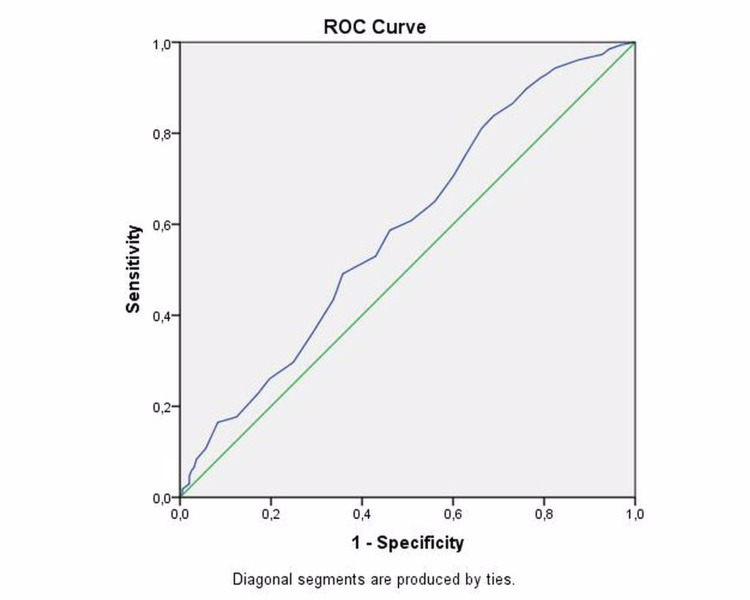
ROC analysis chart. ROC: receiver operator characteristics.

ROC analysis demonstrated that the area under the curve for the model containing age was 0.589 (95% CI 538 to 640) and sensitivity and specificity of the age level for identifying high-risk patients for mortality were 58.7% and 53.9%, respectively, with an optimum diagnostic cut-off value of 80.5 years (Figure 5).

## Discussion

Previous studies reported that the presence of peripheral artery calcification is associated with low bone volume and coronary artery calcification [13,14]. Lower extremity calcification is also found associated with mortality [15]. However, there is only one study reporting the effect of femoral artery calcification on mortality risk in patients with hip fracture [16]. In this study, we investigated the relationship between femoral arterial calcification identified on hip radiographs and all-time mortality of patients with hip fracture aged >65 years. We found that the presence of femoral arterial calcification was independently associated with a poor first month, first year and overall survival.

Previous studies have shown that arterial calcification is an active and complex process in which the vascular smooth muscle cells are involved and synthesize a group of bone-associated proteins [17,18]. Calcification of lower extremity was classified as intimal, medial and mixed type [10,11]. The intimal calcification is associated with atherosclerosis while medial calcification directly increases arterial stiffness and is widespread in persons with metabolic disorders such as diabetes [19]. Clinical consequences of calcification in cardiovascular practice include heart failure, ventricular hypertrophy, diastolic dysfunction, valvular sclerosis and stenosis, and hypertension [19]. Vascular calcification of lower extremity is also recognized as a significant, independent predictor for cardiovascular events [16]. Rennenberg et al. reported a meta-analysis which included 218,080 subjects after a mean follow-up of 10.1 years [5]. In this analysis, the authors found that the presence of calcification in any arterial wall is associated with a 3-4-fold higher risk of mortality and cardiovascular events. Another study, Huang et al. investigated the association between lower extremity arterial calcification and clinical outcomes in patients with symptomatic peripheral artery disease which included Eighty-two patients with symptomatic peripheral artery disease [16]. In that study, they reported that lower extremity arterial calcium score are associated with disease severity and outcomes, including amputation and all-cause mortality, in patients with symptomatic peripheral artery disease. In this study, the presence of calcification in the femoral artery was determined by hip radiographs. We found a significant relationship between femoral arterial calcification and survival of patients with hip fracture older than 65 years.

We found that patients with femoral arterial calcification had two times higher postoperative surgical site infection than patients without femoral arterial calcification. This situation has been described for the first time in the literature.

Articles in the current literature have shown that older age, pre-existing medical conditions, time to surgery, and higher ASA score are the main prognostic factors affecting mortality of patients after proximal femur fractures [20-22]. Rosso et al. reported a large cohort study which included a total of 1,448 consecutive patients with 1558 proximal femoral fractures (55 bilateral) [22]. In that study, they found that age, gender and number of co-morbidities influenced both early and late mortality in patients affected by proximal hip fractures. Additionally, in this study, they reported that patients aged under 74 years had a decreased mortality. In our study, we found a significant correlation between age and survival of patients with hip fracture, especially patients older than 80 years. In our study, we also found a significant relationship between mortality and patients with treated hemiarthroplasty. This relationship may depend on the operation time or the use of cement which data were not available in our study.

This study had some limitations. Since there is a large series of patients, values that are not clinically important can be found to be significantly different between the groups. Many clinical factors may be associated with mortality such as end-stage kidney disease, which we did not assess in this study. The femoral arterial calcification was only evaluated with hip radiograph, CT scan examination is required for formal examination of the calcification and even scoring. Lastly, operation time and use of cement in hemiarthroplasty group were not investigated in this study.

## Conclusions

We found age, treatment modality (hemiarthroplasty), and presence of femoral arterial calcification, to be independent predictors of mortality in elderly patients with hip fracture. The presence of femoral arterial calcification on the affected side of hip was the independent radiological parameter associated independently with a poor first month, first year and overall survival, as patients had 1.5 times higher mortality rate. Additionally, we found a significant correlation between age and survival of patients with hip fracture, especially patients older than 80 years.
